# Airway management in a patient with nuchal, interspinous, and flavum ligament rupture by a sickle: a case report

**DOI:** 10.1186/s13256-016-0957-9

**Published:** 2016-06-13

**Authors:** Kotaro Sorimachi, Yuko Ono, Hideo Kobayashi, Kazuyuki Watanabe, Kazuaki Shinohara, Koji Otani

**Affiliations:** 1 Emergency and Critical Care Medical Center, Fukushima Medical University Hospital, 1 Hikarigaoka, Fukushima, 960-1295 Japan; 2 Department of Anesthesiology, Ohta General Hospital Foundation, Ohta Nishinouchi Hospital, 2-5-20 Nishinouchi, Koriyama, Fukushima 963-8558 Japan; 3 Department of Orthopedics Surgery, Fukushima Medical University Hospital, 1 Hikarigaoka, Fukushima, 960-1295 Japan

**Keywords:** Case report, Hypovolemic shock, In-line cervical immobilization, Penetrating neck injury, Rapid-sequence intubation technique

## Abstract

**Background:**

Penetrating neck injury is an important trauma subset but is relatively rare, especially when involving the posterior cervical column. Rupture of the neck restraints, including the interspinous and flavum ligaments, can create serious cervical instability that requires special consideration when managing the airway. However, no detailed information regarding airway management in patients with profound posterior neck muscle laceration and direct cervical ligament disruption by an edged weapon is yet available in the literature.

**Case presentation:**

A 63-year-old Japanese man attempted to cut off his head using a sickle after drinking a copious amount of alcohol. On admission, his posterior vertebral column was grossly exposed and the lacerated tissues were actively bleeding, resulting in severe hypovolemic shock. We used a rapid-sequence intubation technique with direct laryngoscopy while manual in-line stabilization of his head and neck was maintained by several people. Surgical exploration revealed nuchal, interspinous, and flavum ligament rupture between his fourth and fifth cervical vertebrae, but no injury to the great vessels was present. The major source of bleeding was a site of oozing from his trapezius and splenius muscles. After surgical hemostasis, wound repair, and subsequent intensive care, our patient was discharged home without any neurological sequelae.

**Conclusions:**

Deficits of the neck restraints can cause cervical spine subluxation and dislocation secondary to neck movement. Thus, the key to successful airway management in such a scenario is minimization of neck movement to prevent further neurological impairment. We successfully managed an airway using a conventional but trusted endotracheal intubation strategy in a patient with multiple traumas and a suspected spinal cord injury. This case also illustrates that, even when great vessel injury is absent, severe hypovolemic shock may occur after profound neck muscle laceration, requiring immediate surgical intervention.

## Background

The neck contains vital organs, including large vessels, the trachea, and nerves, within a confined space. A penetrating neck injury (PNI) can directly jeopardize airway patency, oxygenation, and the circulatory status. Definitive airway management and surgical hemostasis are key lifesaving treatments in this important trauma subset.

PNI is not commonly seen in developed countries, especially a PNI involving the posterior neck [1–3]. Cervical musculoligamentous structures, including the nuchal, interspinous, and flavum ligaments, play a significant role in maintaining spine stability [4–6]. Deficits of these restraints can cause cervical spine subluxation and dislocation secondary to neck movement [4–6], requiring special anesthetic consideration. To the best of our knowledge, however, no detailed information regarding airway management in patients with direct cervical ligament rupture by an edged weapon is available in the literature. Therefore, we present our experience with a case of self-inflicted profound posterior neck muscle laceration involving nuchal, interspinous, and flavum ligament disruption.

## Case presentation

**Fig. 1:**
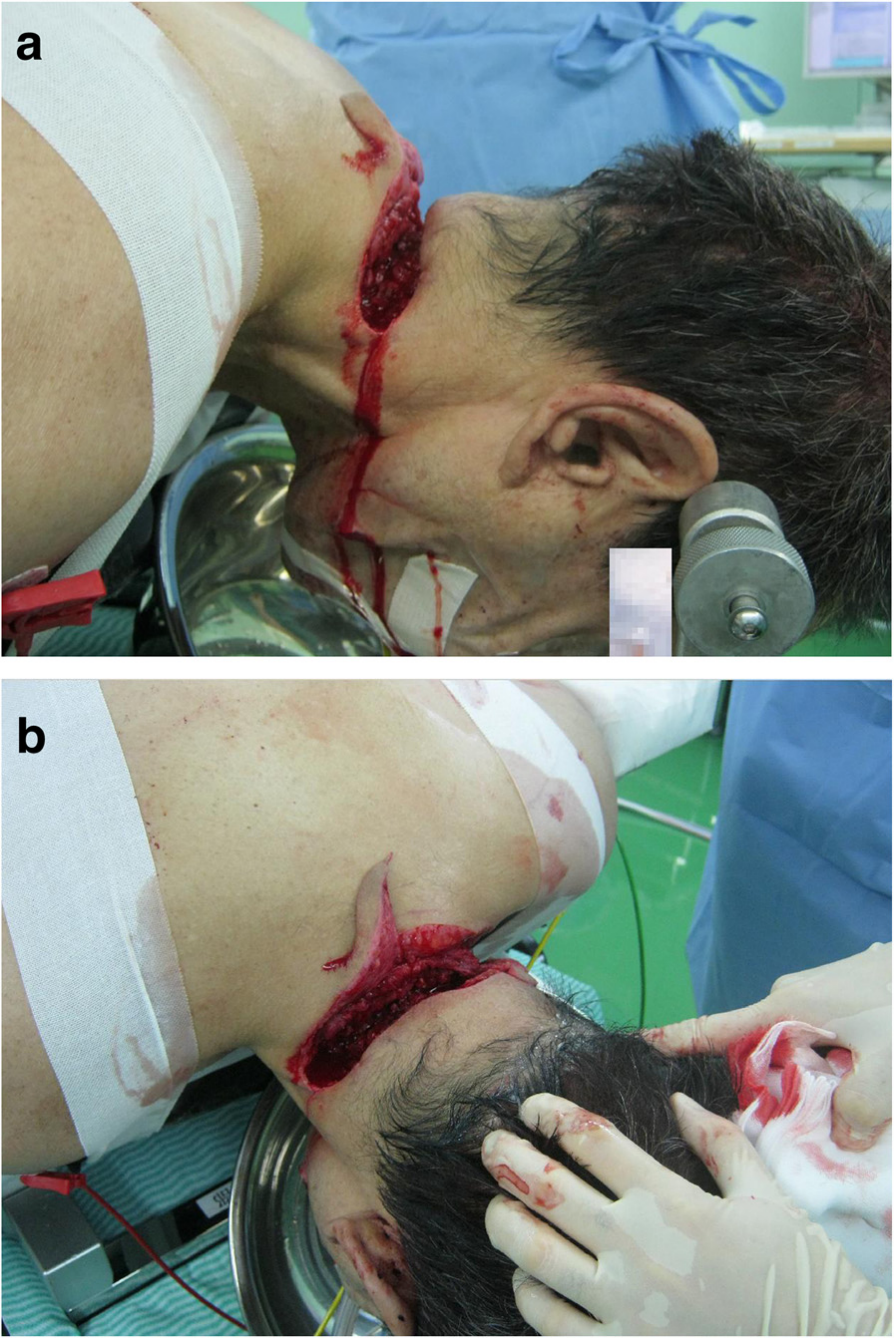
Self-inflicted penetrating posterior cervical column injury by a sickle. The posterior vertebral column was grossly exposed, and the lacerated soft tissues bled actively. **a** Lateral view and **b** craniad view

A 63-year-old Japanese man attempted to cut off his head with a rusty sickle immediately after drinking a copious amount of alcohol. On admission to our emergency department (ED), he was in the supine position and manually immobilized by several paramedics. A physical examination revealed gross exposure of his posterior vertebral column and active bleeding from lacerated tissues (Fig. 1a, b; image obtained in the operating room). Manual pressure hemostasis was provided but was unsuccessful, and our patient developed serious hypovolemic shock. His initial vital signs recorded in our ED were as follows: body temperature, 34.0 °C; heart rate, 140 beats/min; blood pressure, not measurable (the femoral artery was faintly palpable but the radial artery was not); and respiratory rate, 30 breaths/min. He was restless, and his conscious level was 9 on the Glasgow Coma Scale (E2V2M5). His extremities were cool and wet, but no trauma was evident. Our patient was in obvious distress, preventing us from performing a detailed neurological examination. His breath smelled of alcohol. He was lean and did not have a short neck or micrognathia; he showed no signs of restricted mouth opening. The remainder of the physical examination, including assessment of his thorax, abdomen, and pelvis, was normal. He had no history of medication or allergies.

**Fig. 2:**
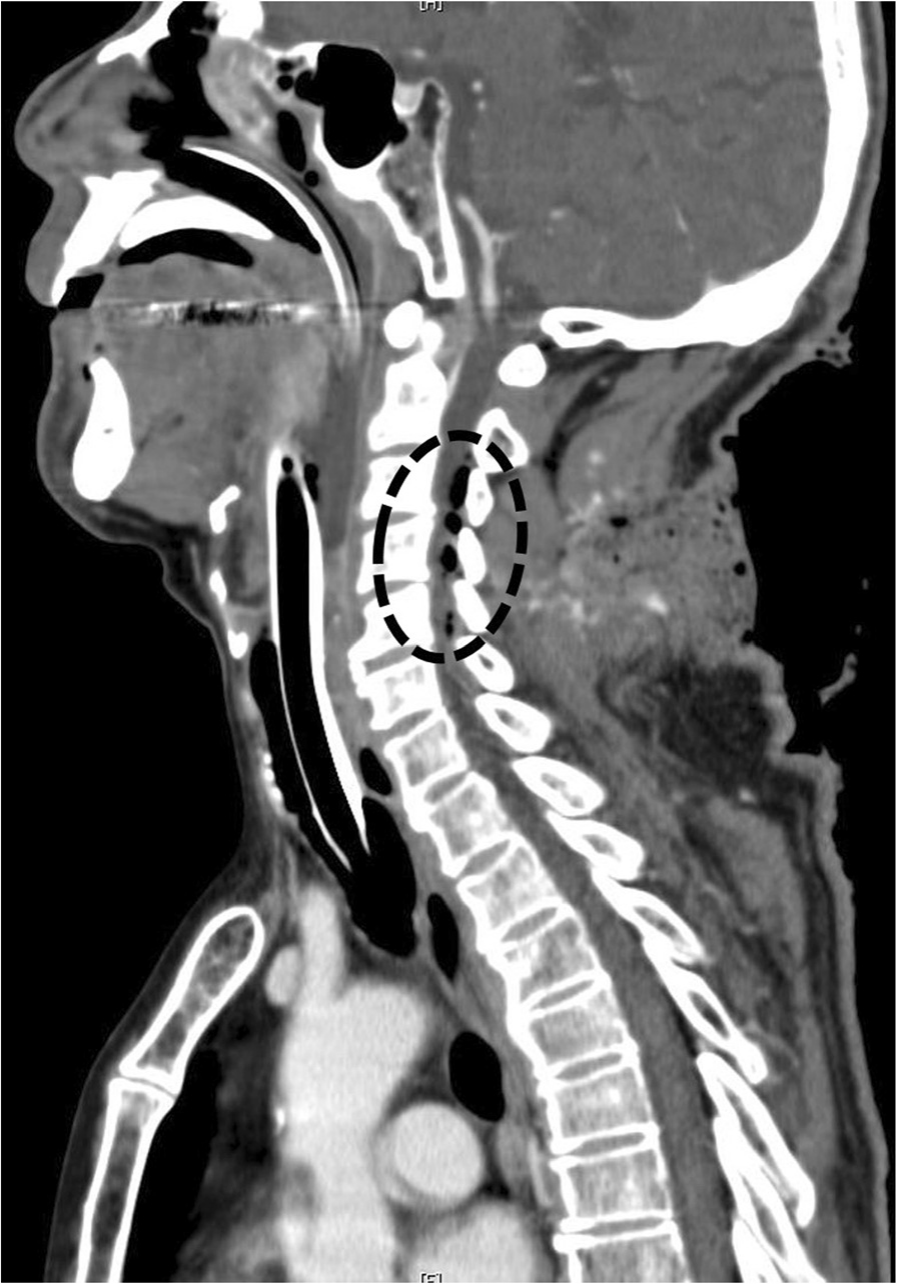
Computed tomography scan showing air in the spinal canal. The *black circle* indicates air in the spinal canal, suggesting dura mater perforation

The need for immediate definitive airway management and surgical hemostasis was apparent. While several people maintained manual in-line stabilization and pressure hemostasis of his head and neck, anesthesia was induced in our ED. Alternative ventilation and intubation equipment, including a supraglottic airway device, video laryngoscope, and surgical airway device, was set up, and we performed rapid-sequence intubation (RSI) with intravenously administered fentanyl (1 μg/kg), ketamine (1 mg/kg), and rocuronium (1 mg/kg) using a conventional laryngoscope. Direct laryngoscopy provided a Cormack–Lehane grade 1 view and revealed neither airway distortion nor edema. An endotracheal tube (inner diameter, 7.0 mm) passed his vocal cords easily. Computed tomography revealed air in his spinal canal, suggesting that the dura mater was perforated (Fig. 2).

**Fig. 3:**
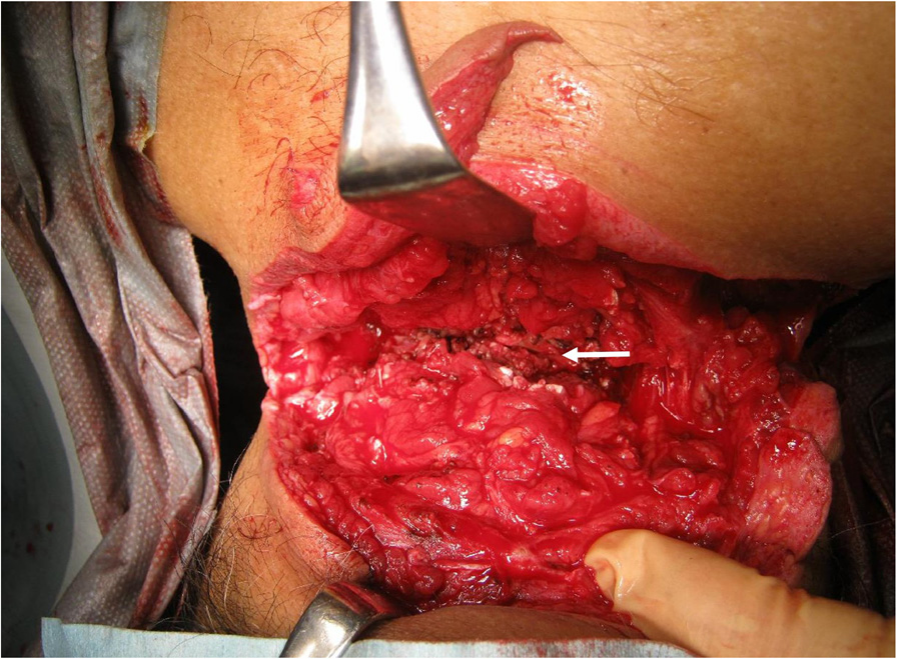
Operative findings. The nuchal, interspinous, and flavum ligaments between the fourth and fifth cervical vertebrae were ruptured. Major cerebrospinal fluid leakage and vertebral artery injury were absent. The *white arrow* indicates the perforated flavum ligament

Surgical exploration revealed laceration of the interspinous and flavum ligaments between his fourth and fifth cervical vertebrae, and the dura mater was exposed (Fig. 3). Fortunately, neither major cerebrospinal fluid leakage nor vertebral artery injury was present. Therefore, surgical repair of the dura mater and large vessels was not required. The major source of bleeding was oozing from lacerations of the trapezius and splenius muscles of his neck; bleeding from both sites was surgically controlled. The facet joints were also intact, which convinced us that cervical stability could be ensured if wound repair and external fixation were provided. After copious irrigation, fascia and soft tissue repair, and cervical collar installation, our patient was admitted to our intensive care unit where he was maintained on controlled ventilation. An examination using a flexible fiberscope the following day revealed neither airway distortion nor edema, allowing successful extubation. His vocal cord movement was also normal. A neurological examination revealed no deficits. He was treated with intravenously administered cefazolin for 3 days to prevent surgical site infection. On day 5, he was transferred to our psychiatric department. The cervical collar was removed on day 14. After further psychiatric evaluation and treatment, he was discharged home and returned to normal activity. At his outpatient follow-up appointment 6 months later, he was neurologically intact and had no neck deformity, including kyphosis or torticollis.

## Discussion

We have herein reported our airway and surgical management experiences in a case of devastating soft tissue disruption of the posterior neck. Direct rupture of neck restraints, including the nuchal, interspinous, and flavum ligaments, leads to severe spinal instability [4–6]. Therefore, the key to airway management is to minimize movement of the cervical spine. This case also illustrates that PNI of the posterior cervical neck requires immediate surgical treatment even when great vessel injury is absent.

This case illustrates the considerations required when managing the airway of patients with disrupted posterior neck restraints. According to the porcine trauma model described by Oxland *et al*. [4], complete injury to both the interspinous and flavum ligaments increases flexion motion by 180 %. Using human cadaver models, Richter *et al*. [5] also showed that interspinous and flavum ligament injury increases spinal instability in flexion/extension, axial rotation, and lateral bending. Thus, all neck movements, especially flexion, must be avoided when managing an airway. A variety of different endotracheal intubation (ETI) methods has been used to attain this purpose. Direct laryngoscopy with manual in-line cervical immobilization is a traditional ETI strategy in patients with multiple traumas who are at risk for or have spinal cord injury [7, 8]. We used this conventional, trusted technique and achieved a satisfactory outcome. Another attractive choice is ETI using video laryngoscopy. At least in planned anesthesia, video laryngoscopy can reduce the movement of the cervical spine during ETI compared with conventional laryngoscopy [9–11]. Use of a bougie as an aid during video laryngoscopy may further reduce movement [11]. The anesthesiology literature also reveals that video laryngoscopy can offer a better glottic view than conventional laryngoscopy in patients with limited neck movement [12]. Nevertheless, whether video laryngoscopy can reduce neck movement in the emergency setting has not been elucidated [9–11]; whether it is more successful in trauma subsets of patients is also unclear [12]. Nasotracheal intubation is also known to decrease motion of the spinal segments compared with oral intubation [13], and fiber-optic intubation has been considered the gold standard technique with which to manage patients with restricted neck movement in planned anesthesia. However, nasotracheal intubation is less successful when used in the emergency setting [1] and can cause serious complications, including nasopharyngeal bleeding and retropharyngeal perforation [8]. Fiber-optic intubation is sometimes time-consuming, and the technique requires extensive training [7]. It is well known that fiber-optic intubation has a high failure rate when this technique is used in the ED [14]. Valero *et al*. [7] successfully used a laryngeal mask to manage a patient with a drill bit penetrating his spinal canal. They insisted that use of a laryngeal mask was highly effective in minimizing cervical spine movement and providing adequate ventilation during the operation. Because our patient had drunk alcohol immediately before his suicide attempt, we could not apply this technique in the present case.

The use of RSI is controversial for airway management in patients with PNI [1–3] because such patients likely have an edematous and/or distorted airway, making both manual ventilation and ETI difficult. Therefore, we adequately prepared back-up ventilation and an intubation strategy before induction of anesthesia. However, at least in our case, there was no airway deformity and RSI was highly effective in facilitating safe ETI. The cough reflex may have abolished the cervical spine stability, and the cardiovascular response associated with ETI might have increased bleeding from the deeply lacerated posterior neck muscle. In this case, RSI successfully prevented both of these important adverse events and provided a satisfactory intubation condition. Logically thinking, PNI of the posterior neck is less likely to cause upper airway edema. Thus, RSI may be the most reasonable approach in patients with a penetrating posterior cervical injury.

This case also illustrates that deep laceration of the posterior cervical column can result in profound hypovolemic shock, even when great vessel injury is absent. The blood supply to the posterior cervical muscle is very rich, and manual hemostatic pressure may not be sufficient, as seen in this case. Therefore, immediate surgical intervention is vital in patients with profound posterior PNI.

## Conclusions

We have reported our airway management experience in a case of direct nuchal, interspinous, and flavum ligament rupture by a sickle. Regardless of the technique used, achieving maximal stability of the cervical spine is vitally important in such a scenario because neck movement secondary to ETI can result in cervical spine dislocation and further neurological deficits. We successfully managed an airway using direct laryngoscopy with manual in-line cervical immobilization by several people, which is a trusted strategy of ETI in patients with multiple traumas who have an established or suspected spinal cord injury. Even when great vessel injury is absent, surgical intervention must be initiated as soon as possible in patients with profound posterior PNI because the blood supply to the posterior cervical muscle is very rich and manual hemostatic pressure may not be sufficient.

## Abbreviations

ED, emergency department; ETI, endotracheal intubation; PNI, penetrating neck injury; RSI, rapid-sequence intubation
